# Nursing Management in Pediatric Intensive Care: From Survival to Family-Centered Recovery

**DOI:** 10.3390/children13101374

**Published:** 2026-10-10

**Authors:** Yujiro Matsuishi

**Affiliations:** Women’s Health Nursing and Midwifery, Nursing Science and Engineering, Institute of Medicine, Nursing Science and Engineering, University of Tsukuba, Tsukuba 305-8575, Japan; matsuishi.yujiro.xa@alumni.tsukuba.ac.jp

## 1. From Survival to Family-Centered Recovery

Improved survival has changed the clinical purpose of pediatric intensive care. Discharge from the pediatric intensive care unit (PICU) remains an essential outcome, but it does not describe the health of a child or family in the months that follow. The pediatric post-intensive care syndrome (PICS-p) framework places physical, cognitive, emotional, and social recovery within the child’s developmental and family context [1]. This broader view has practical consequences at the bedside. The ICU Liberation framework and the ABCDEF Bundle bring pain control, ventilation, sedation, delirium, mobility, and family engagement into a coordinated model of care. In a pediatric multicenter collaborative, greater bundle use was associated with lower mortality, although improvement was not uniform across all outcomes [2]. Because nurses assess these domains repeatedly and coordinate much of their delivery, nursing practice is central to recovery-oriented critical care.

The evidence base has not yet caught up with this expanded responsibility. Units use different measures, often in only one language or health system; family outcomes are assessed inconsistently; and follow-up commonly ends at discharge. Staffing, documentation, training, and local routines also determine whether an intervention can be sustained. Together, these factors affect who is recognized as needing help and whether bedside care can be linked to later outcomes.

The five papers in this Special Issue, “Nursing Management in Pediatric Intensive Care,” address different points in this pathway: clinical and family-reported measurement, parental psychological responses, family-centered services, and a routine neonatal procedure with a limited evidence base. They show that nursing management extends beyond the organization of bedside tasks. It also determines what is assessed, whose knowledge informs care, and which practices are retained.

## 2. What This Special Issue Adds

### 2.1. Measurement for Clinical Care and Comparative Research

Reliable measurement makes a clinical problem visible and allows practice to be compared over time. The SOS-PD delirium component (Sophia Observation withdrawal Symptoms-Pediatric Delirium) and the Cornell Assessment of Pediatric Delirium both show that structured bedside assessment can make delirium screening feasible in routine pediatric critical care [3,4]. Japanese validation studies of the SOS-PD further support its use for iatrogenic withdrawal syndrome and pediatric delirium and show that family observations of altered behavior can contribute meaningfully to delirium recognition [5,6]. The clinical lesson is broader than any single scale. Standardization need not exclude family knowledge; a well-designed assessment can give observations from nurses and families a defined place in routine surveillance.

The same principle applies to the experience of care. The MPOC-20 (Measure of Processes of Care) and EMPATHIC (EMpowerment of PArents in THe Intensive Care) instruments were developed to measure family-centered processes of care and parental satisfaction with pediatric intensive care [7,8]. Cross-cultural validation studies, including Arabic MPOC-20 validation, Spanish EMPATHIC-30 validation, and Japanese EMPATHIC-30 validation, illustrate why such instruments cannot be assumed to perform identically in another language or health system [9,10,11]. Cross-cultural validation must test whether the construct remains meaningful locally while retaining enough consistency for comparison.

PICUs also need a way to connect measurements taken at different stages of recovery. Symptom scales guide decisions during admission; family-reported measures describe communication, participation, and support afterward. Agreed assessment points and compatible outcomes would improve comparison without imposing unreasonable burdens. The aim is a framework in which cultural differences can be described rather than hidden.

### 2.2. Families as Partners in Recognition and Recovery

Critical illness affects parents differently, even when their children have similar clinical courses. Systematic review evidence shows that psychological outcomes after PICU admission are shaped by multiple child, illness, and family factors rather than by clinical severity alone [12]. A pilot study in this Special Issue similarly examined how parental personality traits may relate to post-traumatic stress symptoms and anxiety [13]. Such findings should not be used to classify parents. They reinforce a practical point: parental distress may need to be assessed repeatedly as circumstances change.

Qualitative work in neonatal intensive care (NICU) shows that maternal stress and coping are shaped by pregnancy and birth experiences, the NICU environment, emotional regulation, social withdrawal, and spiritual coping [14]. Systematic review evidence also supports the importance of parent-level risk factors after neonatal intensive care [15]. The same hospitalization can therefore be understood and remembered in markedly different ways. Clinical severity or a single distress score will not capture that experience fully.

The intervention literature provides limited guidance on how to respond. A recent scoping review found substantial variation in PICU family-centered interventions and outcomes; only four of 38 studies involved parents in design [16]. This weakens relevance and reproducibility. Parents can identify when contact is helpful, which forms of follow-up are feasible, and whether an outcome reflects meaningful recovery. Their involvement is a methodological requirement, not a symbolic gesture.

### 2.3. Examining Routine Practice and De-Implementation

Nursing management also determines how routine practices are introduced, maintained, and questioned. Rossi et al. reviewed rectal stimulation in premature and full-term newborns, a practice for which no shared guidance exists [17]. The search identified 62 records, but only two studies met the inclusion criteria. Both came from the same research group and involved extremely preterm infants. The evidence was insufficient to establish effectiveness, safety, clinical indications, or a standardized technique.

Quality improvement cannot focus only on adopting new interventions. Familiar practices may persist before their benefits and harms have been adequately studied. Because nurses repeatedly perform or observe these procedures, they often see variation in indications, technique, and burden first. Organizations need a route for staff to report such uncertainty and for clinical leaders to decide whether a practice warrants study, restriction, or withdrawal.

De-implementation requires the same discipline as implementation. Reviews of low-value care emphasize that reducing or withdrawing established practices requires explicit methods, stakeholder engagement, and outcome monitoring [18]. Removing a familiar procedure without documenting its purpose or providing an alternative can create new variation. Teams need to record why it is used, define plausible benefits and harms, explain changes to families, and monitor outcomes after withdrawal. Nursing quality depends on the ability to question established care as well as to introduce new practice.

## 3. Research and Service Priorities

### 3.1. Establish Longitudinal, Family-Centered Follow-Up

Most studies still provide a single-center snapshot of recovery. Larger multinational cohorts are needed to follow children and families from admission through the return home, to school, and to community life. A shared core of outcomes should extend beyond symptoms to child health, family functioning, participation, and service access. Studies should also record the timing and adequacy of formal and informal support, which can alter the consequences of distress.

Follow-up begins with planning before discharge. Repeated assessment can distinguish resolving distress from problems that persist or emerge later. Families facing language, geographic, financial, or digital barriers are easily lost, producing reassuring results from an unrepresentative sample. Core outcomes provide a common structure; developmental stage, family composition, previous health, and local service access explain variation within it. Families should help select measures and interpret clinically meaningful change.

### 3.2. Test Nurse-Led, Risk-Responsive Support

Describing risk is no longer enough. Research must test what support changes outcomes, for whom, and when. Prior mental illness, social isolation, prolonged critical illness, repeated admission, or marked distress may justify additional contact. These indicators should open access to support rather than become fixed labels. Nurses can coordinate clear information, repeated assessment, brief psychological support, peer contact, and specialist referral.

Support intensity should change over time. Some families need reassurance and a reliable contact; others require repeated screening and active referral. Stepped programs need evaluation in culturally adapted, co-designed multicenter trials, with reporting detailed enough for replication [16]. Evaluation should include uptake, family burden, referral completion, cost, and reach as well as symptom improvement.

### 3.3. Create an Interoperable Foundation for Nursing Data

PICUs collect extensive nursing data on symptoms, mobility, communication, and family participation, but differences in definitions and documentation limit comparison. Making these data interpretable across sites requires a consensus dictionary, validated measures, standardized terminology, and metadata describing how each observation was made. Standardized electronic nursing data can characterize care and its relationship with patient outcomes [19]. Resulting datasets should follow FAIR principles: findability, accessibility under appropriate conditions, interoperability, and reusability [20].

Predictive modeling is a later step. Algorithms trained on poorly defined or locally specific data will reproduce those limitations at scale. External validation, bias monitoring, and clinical oversight are necessary before model outputs influence care. Future critical-care nursing data platforms should therefore use culturally adapted measures while remaining compatible with data from other languages, countries, and resource settings.

Standardization has limits. A common variable is difficult to interpret without information on staffing, workflow, assessment frequency, and missingness; these contextual elements belong in the dataset. Families and clinicians also need a role in decisions about access, secondary use, and predictive models. Data can support learning only when the people represented have reason to trust how those data will be used.

## 4. Conclusions

The papers in this Special Issue show that nursing management in pediatric intensive care encompasses symptom assessment, the integration of family knowledge into care, psychological support, and critical evaluation of routine procedures. Decisions in each of these areas can influence not only care during admission but also the subsequent recovery of children and their families.

Future work should establish longitudinal follow-up that links inpatient assessment with outcomes after discharge, test nurse-led interventions co-designed with families, and develop nursing data infrastructures that support comparison across institutions. Greater consistency in measures and data should be pursued without losing essential clinical context, including developmental stage, family circumstances, and the organization of care. Together, these approaches can clarify how nursing practice shapes long-term recovery and support continuous improvement in the quality of pediatric intensive care.

## Data Availability

No new data were created or analyzed in this editorial. Data sharing is not applicable.
